# The impact of climate change-related disasters on mental health and epigenetics: a narrative review

**DOI:** 10.3389/freae.2024.1464276

**Published:** 2024-10-14

**Authors:** Emma Antonina Roe, Engluy Khov, Hannah Kim, Beth Demelo, Savannah Zavitz, Lei Cao-Lei

**Affiliations:** Faculty of Health Sciences, University of Ottawa, Ottawa, ON, Canada

**Keywords:** climate change, disaster, mental health, epigenetics, prevention and intervention

## Abstract

Climate change has contributed to an increase in the frequency and intensity of natural disasters such as droughts, wildfires, hurricanes, and floods, leading to both immediate physical harm and long-term mental health consequences. Survivors often experience psychological distress, including anxiety, depression, and post-traumatic stress disorder (PTSD), as a result of these traumatic events. This narrative review explores the intersection of climate change-related disasters, mental health outcomes, and epigenetic modifications. Specifically, we summarize recent findings on how environmental stressors influence both mental health and epigenetic changes, such as DNA methylation. Emerging evidence suggests that epigenetic mechanisms, particularly DNA methylation, could mediate the effects of climate change-related stress on mental health, potentially contributing to the onset of mental disorders like depression, panic disorder, autism spectrum disorder, and attention deficit hyperactivity disorder. We also discuss other epigenetic mechanisms, such as histone modifications and non-coding RNAs, and emphasize the importance of longitudinal studies to capture the dynamic nature of epigenetic changes over time. Finally, we propose future research directions to deepen our understanding of the complex relationships between climate change-related disasters, mental health outcomes, and epigenetic mechanisms, which will pave the way for more effective mental health interventions and policy integration.

## Introduction

Climate change has become an overwhelming issue over the last few decades. In 2023, the Intergovernmental Panel on Climate Change (IPCC) released its Sixth Assessment, report, declaring that the global climate is in a code red status and that “we are way off track” on reducing its impact. Consequently, there has been an increase in natural disasters, such as earthquakes, hurricanes, and floods, among other similar events. Those affected will often face physical or emotional damage through injury, death, relocation, and loss of livelihood (Masson-Delmotte et al., 2021).

Mental health is integral to a person’s health and can be altered due to stressful life events, such as climate change-related disasters. Climate change directly and indirectly impacts the frequency and intensity of these natural disasters, causing immediate physical damage and long-term effects on mental health and wellbeing. Survivors often experience stress, anxiety, depression, and post-traumatic stress disorder (PTSD) due to the psychological trauma associated with these events (Saeed and Gargano, 2022).

Epigenetics refers to heritable changes in gene expression that do not alter the underlying DNA sequence but can profoundly affect how genes are turned on or off, influencing an individual’s development, physiology, and disease susceptibility (Bird, 2007). These modifications, such as DNA methylation, histone modification, and non-coding RNAs, regulate gene expression by altering chromatin structure or modifying RNA stability, enabling genes to respond to environmental stimuli (Allis and Jenuwein, 2016). Epigenetic changes are also reversible, offering therapeutic potential through drugs like DNMT and HDAC inhibitors that target aberrant epigenetic states linked to diseases (Singh et al., 2022). Understanding these mechanisms provides crucial insights into how environmental factors, including climate change-related disasters, impact health at the molecular level. As research progresses, it highlights the dynamic role of the epigenome in shaping health risks and offers new avenues for both public health interventions and novel treatments.

The objective of this narrative review is to explore the intersection of climate change, natural disasters, mental health, and epigenetics. Specifically, we summarize recent findings on the impact of common climate change-related disasters on both mental health and epigenetic changes. Additionally, we propose future research directions and offer perspectives to deepen the understanding of the complex relationships between climate change-related disasters, mental health outcomes, and epigenetic mechanisms.

## Climate change and related disasters

Climate change refers to significant and long-term changes in Earth’s temperature, precipitation patterns, and atmospheric conditions, driven largely by human activities such as burning fossil fuels, deforestation, and industrial processes (IPCC, 2018; Nema et al., 2012). These activities have accelerated the accumulation of greenhouse gases like carbon dioxide and methane in the atmosphere, leading to global warming—a phenomenon that disrupts weather patterns and increases the frequency and severity of natural disasters (Masson-Delmotte et al., 2021). Natural disasters, which include sudden and severe events caused by environmental factors, pose significant threats to human health, safety, and wellbeing (Alexander, 2018). The increase in global temperatures has resulted in more frequent and severe disaster events, including droughts, storms, extreme heat, and heavy rainfall. In the following sections, we provide an overview of several common types of these disasters.

1) Droughts are prolonged periods of deficient rainfall exacerbated by rising temperatures and shifting precipitation patterns (Trenberth et al., 2014). They have severe consequences for agriculture, water supply, and ecosystems, impacting food security and livelihoods (He et al., 2019). Drought can impact respiratory health, mental health, and illnesses related to exposure to toxins, food/water security, rates of injury and infectious diseases (including food-, water- and vector-borne diseases) (Yusa et al., 2015). 2) Wildfires are exacerbated by climate change due to higher temperatures, drier conditions, and prolonged droughts (Di Virgilio et al., 2019). For instance, the devastating 2019-2020 Australian bushfires burned over 18.6 million hectares, causing substantial loss of life and biodiversity, underscoring climate change’s impact on wildfire behavior (Haque et al., 2021). 3) Extreme precipitation events, intensified by climate change, result from warmer oceans holding more moisture, leading to more intense storms like hurricanes, typhoons, and cyclones (Trenberth, 2007). These storms bring heavy downpours and powerful winds, causing catastrophic flooding, landslides, and infrastructure damage, resulting in significant human and economic losses (Gordon and Young, 2021). 4) Floods occur when water submerges dry land, often due to heavy rainfall, rapid snowmelt, or storm surges from tropical cyclones. The 2010 floods in Pakistan, affecting over 20 million people, illustrate the widespread damage to infrastructure and displacement of communities caused by climate-induced flooding (Jacquet et al., 2016). 5) Ice storms, influenced by warmer ocean temperatures and altered precipitation patterns, can lead to heavy ice accumulation, resulting in widespread power outages, infrastructure damage, and hazardous travel conditions, as demonstrated during the 1998 Quebec ice storm (Germain and Martin, 2011). 6) Earthquakes, primarily caused by tectonic forces, can be indirectly influenced by climate change through factors like droughts, increased rainfall, melting glaciers, ice caps, and human activities such as fracking (Sadhukhan et al., 2022).

It is crucial to continuously monitor the trends in the frequency and intensity of climate change over time to inform public health and further research into the impact of climate change on the human population.

## The effect of climate change-related disasters on mental health

In the following section, we summarize recent publications that investigate the impact of various climate change-related disasters on mental health.

### Drought and heat waves

A systematic review conducted by Thompson et al. (2018) describes the mental health effects of high ambient temperature and heat waves. It investigates whether heat-related morbidity and mortality are increased among individuals with known mental disorders. This review concluded that high temperatures have a wide range of mental health effects, with the strongest evidence linking them to increased risk of suicide. Similarly, Hanigan and Chaston (2022) examined the impact of drought on suicide rates in rural New South Wales, Australia, and found that drought increases suicide rates among rural males aged 10–29 and 30–49 while decreasing rates among rural females aged 30–49. The exacerbating effects of climate change on these trends highlight the need for targeted interventions such as counseling services, community support networks, financial aid, and public health messaging. Addressing the mental health challenges posed by drought requires multidisciplinary approaches and policy development to enhance community resilience. Furthermore, Varshney et al. (2023) provided a systematic review examining the global mental health outcomes of vulnerable populations affected by natural hazards such as drought and bushfires. They highlighted that such events disproportionately affect these groups, exacerbating pre-existing mental health issues and socioeconomic challenges, leading to increased anxiety, depression, PTSD, and suicidality. This review emphasizes the necessity for tailored mental health interventions and policies that address structural barriers and provide long-term support, promoting resilience and recovery in vulnerable communities. In a similar vein, Luong et al. (2021) investigated the intricate relationship between prolonged drought exposure and mental health among rural communities in New South Wales, Australia, showing that psychological distress initially rises during the early years of drought, but people gradually adapt, leading to decreased distress over time. However, this adaptation does not equate to complete recovery, as overall life satisfaction declines with long-term drought exposure. The study highlights the necessity for continuous support, resilient communities, and proactive drought management to address the persistent and complex mental health challenges posed by drought conditions. Adding to this body of research, Sewell et al. (2024) examined how concurrent heatwaves and droughts significantly increase mental health risks in children, adolescents, and young adults in North Carolina. Their findings showed that these climate events had a clear impact on emergency department visits for mood disorders and suicidality, especially among those in socioeconomically disadvantaged communities. The authors suggest targeted public health interventions and policy measures to enhance community resilience, improve access to resources, and address the social determinants of health to mitigate these impacts.

### Wildfires

The increasing frequency and intensity of wildfires, driven in part by climate change, have profound effects on mental health. Several studies have explored these impacts, revealing a range of psychological outcomes and identifying factors that contribute to mental health challenges following wildfire exposure. For instance, a study conducted by Cowlishaw et al. (2024) analyzed the relationship between Australian bushfires and mental health during the COVID-19 pandemic. Through semi-structured interviews and focus groups, the researchers assessed mental health outcomes following fire exposure. Their findings revealed six broad themes that helped situate the impacts of the pandemic within the context of adjustment and recovery processes after bushfires. This study underscores the compound nature of disasters and their multifaceted effects on mental health. Similarly, Isaac et al. (2023) investigated the impacts of wildfire on anxiety, insomnia, and trauma symptoms across participants from Australia, the United States, and Canada. Their research found that wildfire exposure led to negative outcomes in these areas, with participants in the USA reporting higher severity of symptoms. Moreover, female subjects exhibited higher rates of PTSD compared to their male counterparts, highlighting gender differences in psychological vulnerability to wildfire exposure. Several studies have specifically considered PTSD as an outcome of wildfire exposure. For example, Macleod et al. (2024) assessed levels of anxiety, depression, stress, PTSD, psychological wellbeing, and resilience following exposure to the 2019 Australian bushfires. Their results indicated that certain demographic factors, including sex, finances, and pre-existing mental health diagnoses, influenced mental health outcomes. Approximately half of the participants, regardless of direct exposure to the bushfires, met the clinical cutoff for depression and anxiety, with 35% of those directly affected meeting the PTSD cutoff criteria. This suggests that wildfire exposure has widespread mental health impacts that transcend direct physical harm. In a related study, Mellish et al. (2024) examined the psychological outcomes of direct and indirect exposure to the 2019/2020 Australian bushfires. The researchers found that 25% of the nonclinical group met the PTSD diagnosis cutoff criteria. Interestingly, those indirectly exposed exhibited higher rates of maladaptive and avoidant coping strategies and lower rates of posttraumatic growth. Furthermore, individuals indirectly exposed reported similar levels of distress as those directly exposed, indicating that even indirect experiences of wildfire can lead to significant psychological distress. The impact of wildfires on vulnerable populations has also been a focus of recent research. Saberi et al. (2023) analyzed the impact of the Northern California wildfires on people with HIV (PWH) and the clinicians providing care to them. The study reported heightened rates of anxiety, hypervigilance, stress, depression, sleep disturbances, and mental health-related coping strategies among PWH following wildfire exposure. Additionally, clinicians noted that their practice had been directly impacted by the wildfires for 2 years after the event, highlighting the long-term implications for healthcare providers and patients alike. Moreover, Watts et al. (2023) investigated PTSD rates and precipitating factors following South Australian bushfire exposure. Their study found that PTSD rates declined over time. However, predictive factors for PTSD included fear for one’s life, loss of a loved one to the fires, loss of property, lower education levels, major life stressors, and being female. Notably, relocation following the fires was identified as a significant stressor for PTSD at the 2-year mark of self-reports, indicating that the process of recovery and adaptation continues long after the immediate disaster.

Collectively, these studies highlight the severe and varied mental health impacts of wildfires. Factors such as gender, direct versus indirect exposure, pre-existing conditions, and demographic characteristics influence psychological outcomes. Understanding these factors is crucial for developing effective interventions and support systems to mitigate the mental health consequences of wildfires.

### Hurricane and typhoon

A study conducted by Burrows et al. (2023) investigated the impact of the physical environment, specifically environmental greenness, on psychological distress outcomes following hurricane exposures. The results indicated significant connections between residential environmental greenness and distress levels, with perceived control over their environment being a notable influential factor on the participants. This study underscores the importance of environmental factors in mitigating psychological distress post-disaster. Building on this, Acierno et al. (2006) analyzed the long-term impacts of hurricane exposure on trauma and mental health outcomes through a longitudinal study. Their findings concluded that older age was associated with a lower intensity of PTSD and depressive symptoms following exposure, highlighting the influence of age and recovery stressors on symptom intensity. These results align with the notion that recovery processes and personal attributes significantly impact mental health outcomes after a hurricane. Similarly, Cohen et al. (2023) conducted a study on the rates of depression and PTSD among Houston residents exposed to Hurricane Harvey during the pandemic. They determined that both stressors and traumas affected PTSD, with stressors acting as a primary influence on depressive symptoms. Miller et al. (2023) analyzed suicide mortality rates following Hurricane Florence and found an overall rate of 15.53 deaths per 100,000 people across a 4-year span for those aged ten and above who were affected by the hurricane. Interestingly, suicide mortality rates initially increased among the exposed group but later decreased to match those of the unexposed group. This trend indicates the complex and evolving nature of mental health impacts following hurricanes. In Puerto Rico, Sackey et al. (2023) investigated the influence of hurricane exposure on symptoms of depression, anxiety, and PTSD among teachers and students. The results revealed that experiences of loss, disruption of life, and an objective threat from the disaster were significant factors in determining the severity of mental health symptoms. A notable sex-effect indicated that participants identifying as female experienced more peritraumatic symptoms and measurable anxiety, emphasizing the role of gender in mental health outcomes post-disaster. Short et al. (2023) gathered data on parent-child dyads affected by Hurricane Harvey and assessed parental anxiety and depressive symptoms, children’s emotional distress, and psychosocial tendencies. They found that parental mental health significantly influenced their children’s emotional outcomes, with parental experiences of disruption and loss being predictive of emotional distress in the child. This highlights the intergenerational transmission of trauma and the importance of addressing family dynamics in post-disaster mental health interventions. Further, Wertis et al. (2023) investigated the influence of Hurricane Ida on community substance use, suicidal ideations, stress/anxiety, and bereavement. Data collected using a free Crisis Text Line (CTL) accessed by community members before and after Hurricane Ida revealed that the number of daily conversations through the CTL service peaked post-hurricane exposure. Reports of suicidal ideations, substance use, stress/anxiety, and bereavement were highest in the period after the hurricane, illustrating the widespread and immediate mental health impacts of such disasters. Lastly, Wang et al. (2023) analyzed the outcomes of parental PTSD on children’s mental health following Typhoon Lekima. They found that the trajectory of parental PTSD directly correlated with the child’s symptoms of PTSD and depression, impacting their feelings of safety. This study highlights the possibility of generational outcomes following exposure to natural disasters and underscores the need for family-centered mental health interventions.

In conclusion, these studies provide comprehensive insights into the mental health impacts of hurricanes and typhoons. They emphasize the importance of environmental, demographic, and familial factors in understanding and addressing the mental health needs of affected populations. The findings underscore the necessity for tailored interventions and ongoing support to mitigate the long-term psychological effects of such natural disasters.

### Flood

Brock et al. (2015) examined the impact of prenatal exposure to the 2008 Iowa floods on perinatal depression and maternal wellbeing and found that increased flood exposure during pregnancy was associated with increased depressive symptoms, significantly mediated by peritraumatic distress, and decreased wellbeing. These findings underscore the need for early psychological interventions to address distress in disaster-exposed populations and mitigate long-term mental health impacts. Similarly, Kingston et al. (2019) studied family resilience after the 2013 Alberta flood using longitudinal data. They found that children’s adverse outcomes depended on factors such as genetics and home environment. Resilience was linked to changes in stress biomarkers, including cortisol, immune function, and metabolomics. This study highlights the importance of developing accurate resilience screening tools by comparing cortisol biomarkers, linking needs to interventions, understanding genetic influences, and informing mental health policy. In Germany, Schürr et al. (2023) investigated the short-term and long-term mental health impacts of the 2016 flood in Simbach am Inn on adolescents through a qualitative interview study. They identified key stressors such as family safety concerns, the extent of damage, and disrupted routines, while protective factors included strong family and community support. These findings emphasize the need for comprehensive mental health care, including educational programs, long-term support, and enhanced training for responders to better address adolescents’ mental health needs post-disaster. Turning to Pakistan, Nadeem et al. (2023) addressed the mental and maternal health challenges faced by women in flood-affected communities. They highlighted the increased vulnerability to mental health issues such as PTSD, anxiety, and depression, along with unique maternal health challenges. This underscores the need for integrated, culturally relevant mental health support, mobile health units, and gender-sensitive disaster response strategies. In another study from Pakistan, Mahesar et al. (2024) investigated the profound impact of the 2022 floods on suicidal behavior by analyzing newspaper reports. The high prevalence of suicides, particularly among males and young adults in flood-affected regions, highlights the need for targeted mental health interventions, culturally sensitive approaches, and collaborative efforts between the government and nongovernmental organizations (NGOs) to address the unique psychological and socio-economic stressors faced by these populations. Integrating mental health support into disaster preparedness and response strategies will help mitigate the long-term mental health consequences of future disasters. Longman et al. (2019) examined the mental health impacts of the April 2017 floods in rural New South Wales, Australia. They found significant psychological distress and probable PTSD among individuals, especially among vulnerable groups such as the elderly, young, socio-economically disadvantaged, and Indigenous populations. This study emphasizes the critical role of effective community and organizational responses in disaster situations. It advocates for integrating mental health considerations into disaster planning and policy development to enhance resilience and recovery in flood-affected communities. In Japan, Miyaji et al. (2022) explored the role of cognitive social capital in reducing the odds of developing PTSD among victims of heavy rainfall and flooding. They found that while cognitive social capital uniformly benefits mental health, the effects of structural social capital vary based on age and sex, with elderly women benefiting and elderly men potentially experiencing adverse effects. This study highlights the importance of using social capital interventions to improve mental health resilience after disasters. Finally, Cherry et al. (2023) explained the significant impact of severe weather events, like floods, on the mental health of middle-aged and older adults. They emphasized the role of various factors, such as age, social support, state hope, recovery stressors, and prior lifetime trauma, in predicting mental health outcomes like PTSD, depression, and worry. This study underscores the importance of mental health services, social support systems, and interventions based on hope theory to mitigate the adverse effects of disasters and promote resilience among affected individuals.

Taken together, these studies provide comprehensive insights into the mental health impacts of floods. They highlight the significance of early intervention, resilience factors, community support, and tailored mental health services in addressing the psychological aftermath of flooding. By integrating mental health considerations into disaster response strategies, it is possible to enhance the resilience and wellbeing of affected populations.

### Ice storm

Li et al. (2023) examined the long-term impacts of prenatal maternal stress (PNMS) resulting from the 1998 Quebec ice storm on brain structure and function in young adults. They discovered that individuals exposed to PNMS had larger gray matter volumes and increased functional connectivity in several brain regions compared to non-exposed controls. This study highlights the significant and lasting effects of prenatal stress on brain development, particularly emphasizing the timing of exposure and maternal cognitive appraisal. Cao-Lei et al. (2021) analyzed the impact of PNMS from the same natural disaster on hippocampal volumes in adolescents. Their research revealed significant gene-by-environment interactions, showing that PNMS affects hippocampal development differently in boys and girls, moderated by COMT and BDNF genetic variants. While objective hardship influenced hippocampal volume in both sexes, subjective distress had a sex-specific effect. These findings underscore the importance of considering genetic susceptibility and the nature of prenatal stressors in understanding brain development.

### Earthquakes

Recent studies have highlighted the significant impact of earthquakes on mental health, with increased risks of depression, PTSD, anxiety disorders, sleep disorders, and stress among survivors.

Research on the 2023 earthquakes in Turkey and Syria has provided valuable insights into these effects across different survivor groups. For instance, in a study on university students in Amman, Alfuqaha et al. (2023) investigated PTSD following an earthquake, finding that 26.20% reported extreme PTSD symptoms, coupled with low levels of meaning in life (ML) and social support (SS). Female students were particularly vulnerable to PTSD symptoms and faced challenges in seeking ML and SS. Complementing these findings, another study by Alpay and Aydın (2024) explored the predictive value of early peritraumatic reactions (e.g., dissociation, distress) for PTSD and depression among general survivors in Turkey, identifying dissociation as the strongest predictor for both conditions. Additionally, in Syria, Ataya et al. (2024) reported increased rates of depression, sleep problems, and anxiety disorders following the earthquake, with women being more susceptible to sleep disorders and depression, and young adults more vulnerable to sleep disorders.

Regarding the 2020 Turkey earthquake, Tanrıkulu et al. (2024) found that healthy subjects had a higher prevalence of earthquake-related PTSD compared to schizophrenia patients, with female gender increasing the risk among healthy subjects. This highlights the differential impact of earthquakes on mental health based on pre-existing conditions and demographics.

The long-term mental health impacts of the 2008 Wenchuan earthquake in China have also been extensively studied. For example, Chen et al. (2023a) identified three patterns of social support among survivors, finding that moderate and high support improved quality of life by reducing depressive symptoms. Another study by Chen et al. (2023b) demonstrated that accumulated stressful events exacerbated mental health problems, even with social support. Adding to this, Ma Z. et al. (2023) identified distinct PTSD trajectory groups among survivors, with specific symptoms predicting remission or chronic dysfunction in different groups. Research on the 1976 Tangshan earthquake in China has also provided valuable insights. Lu et al. (2023) revealed that prenatal exposure to earthquake stress increased the risk of depressive symptoms in adulthood, especially among females. Further, Ma W. et al. (2023) observed that earthquake-related experiences did not affect the severity of schizophrenia or PTSD, but older age and marital changes were linked to more severe PTSD symptoms.

Japan has experienced significant mental health impacts from earthquakes and tsunamis. Studies by Hikichi et al. (2023a); Hikichi et al. (2023b) on the 2011 earthquake and tsunami found correlations between housing damage, present bias, and delayed-onset posttraumatic stress symptoms (PTSS) among older adults. Notably, a higher sense of coherence (SOC) improved mental wellbeing in those with minor property damage. Following the 2016 Kumamoto earthquake, Matsuoka et al. (2023) reported that relocating to temporary housing had mixed effects on major depressive episodes, without clear links to PTSD symptoms.

In Korea, a study by Han (Han, 2023) investigated the impact of the 2016 Kyungju earthquake on mental health, finding that residents, particularly women, low-income individuals, and those near the epicenter, had a higher risk of mood disorders post-earthquake. This underscores the importance of considering socioeconomic factors in understanding the mental health impacts of earthquakes.

Salawali et al. (2020) highlighted posttraumatic growth (PTG) in adolescent survivors of the 2018 Palu earthquake, tsunami, and liquefaction in Indonesia. The study emphasized resilience and positive change facilitated by cognitive and acceptance commitment therapies, alongside social support, demonstrating the potential for growth even in the aftermath of disasters.

The combined impact of COVID-19 and earthquakes in Croatia during 2020 has been studied in different cohort groups. During the COVID-19 pandemic and the 2020 Croatia earthquake, Levaj et al. (2023) compared mental health outcomes and healthcare use among patients with severe mental illness, finding that additional care through Community Mental Health Teams (CMHTs) improved social support and healthcare access. In a complementary study, Šagud et al. (2023) reported higher levels of depression, stress, and fear related to COVID-19 and the 2020 Croatia earthquake among psychiatric patients, with individuals with depression and/or anxiety disorders being more vulnerable.

These studies provide valuable insights into the far-reaching impacts of climate change-induced earthquakes on mental health. They emphasize the importance of tailored interventions that address the specific needs of different age groups, genders, and demographic cohorts, highlighting the significance of mental health prevention and ongoing support in the face of traumatic events.

## The effect of climate change-related disasters on epigenetic modification

In the subsequent section, we explore the intricate associations between several climate change-related disasters and epigenetic modifications (Table 1).

**TABLE 1:** Summary of studies investigating the association between climate change-related disasters and epigenetics.

Authors	Exposure/Disaster	Participants	Sample	Methods	Results
Straight et al. (2022)	Maternal exposed to drought in Kenya in 2009	213 children, 104 exposed and 109 non-exposed controls (1.8–9.6 years old)	Saliva	Illumina Infinium MethylationEPIC Beadchip	16 CpG sites were differently methylated, predominantly in metabolism and immune functioning. A CpG mediator, cg03771070 had a significant indirect effect of drought exposure on child body weight
Qiao et al. (2024)	Maternal exposed to drought in Kenya in 2009	213 children, 104 exposed and 109 non-exposed controls (1.8–9.6 years old)	Saliva	Illumina Infinium MethylationEPIC Beadchip	Three epigenetic clocks were seen to be altered: Hannum’s (1st generation), GrimAge (2nd generation) and DNAm based telomere length (DNAmTL) clock (2nd generation). These have implications in chronic diseases and longevity
Xu et al. (2023)	Women exposed to wildfire	479 Australian women, 132 female twin pairs, and 215 of their sisters (40–70 years old)	Blood	HumanMethylation450K Beadchip	The PM_2.5_ from the exposed group was associated with DNA methylation measures. 26 CpGs and 33 DMRs were found associated with inflammatory regulation and platelet regulation
Kello et al. (2023)	Matenal exposure to Hurricane Maria in 2017	32 mother (16 High PNMS group and 16 low PNMS group) (average 31.2 ± 4.8 years) 32 children (26 male and 6 female (average 17.1 ± 4.3 months))	Blood	Illumina Infinium MethylationEPIC Beadchip	47 significant DMPs were identified, with 30 associated with gestational stage. Most DMPs were hypermethylated, clustering on chromosomes 1–4. DNA methylation differences were also linked to maternal mental status and property damage after the hurricane
Cao-Lei et al. (2014)	Matenal objective hardship resulted from 1998 Quebec ice storm	34 children prenatally exposed to Quebec ice storm	T cells	Illumina Infinium Methylation450K Beadchip	Objective hardship was correlated with DNA methylation levels of 957 genes (1,675 CpGs) predominantly related to immune function
Cao-Lei et al. (2015)	Matenal cognitive appraisal resulted from 1998 Quebec ice storm	34 children prenatally exposed to Quebec ice storm	T cells	Illumina Infinium Menthylation450K Beadchip	Cognitive appraisal was associated with 1564 different genes (2872 CpGs) predominantly related to immune function
Wang et al. (2022)	Prenatal Tangshan earthquake stress exposure	Prenatal exposed group (N = 100), Control group (N = 76)	Blood	Bisulfite sequencing	Prenatal earthquake exposure, particularly during the second trimester, was associated with higher NR3C1 methylation levels and impaired working memory. A moderate negative correlation was found between CpG1 methylation and working memory in the second trimester group
Mendoza-Ortega et al. (2021)	Maternal earthquake stress in Mexico City, 2017	83 mother-infant pairs (22 control infants, 24 pregnancies during earthquake and 37 concieved afterwards)	Umbilical cord blood	Quantitative real-time PCR	Newborns exposed *in utero* or conceived after the earthquake had significantly higher mtDNAcn compared to unexposed newborns

### Drought

Straight et al. (2022) used a cohort of children who had been exposed to a drought *in utero* to investigate the long-term epigenetic effects. Through DNA methylation arrays, they discovered 16 CpG differences between the exposed cohort, and the same-sex control siblings. The CpG differences were linked mainly to metabolism and immune system pathways, leading to higher adipositivity in children exposed, as well as decreased immune function. They also found a different CpG mediator known as cg03771070 which was significantly associated with lowered body weight in the exposed group. A recent study by Qiao et al. (2024), suggests that droughts may be able to cause accelerated epigenetic aging *in utero*, due to the stress experienced by the mother. Through blood samples of children who were affected by droughts, and compared against their same-sex unexposed siblings, a positive association with epigenetic age acceleration (EAA) through two epigenetic clocks was found: Hannum’s clock (Hannum et al., 2013), a first-generation epigenetic clock that serves as a lifespan predictor and GrimAge’s clock (Lu et al., 2019), a second-generation clock known for predicting disease risk. A negative association with EAA was found with another second-generation clock: DNA methylation-based telomere length (DNAmTL) clock. The combined effect of these associations leads to increased risk of chronic diseases and loss of genetic longevity due to the stress experienced by the mothers during a drought.

### Wildfire

A recent study has identified biological markers indicating changes to the human epigenome following wildfire smoke exposure. A research group (Xu et al., 2023) studied the associations between long-term exposure to wildfire related-PM2.5 and blood DNA methylation in 479 Australian women, including 132 female twin pairs and 215 of their sisters. Using the HumanMethylation450K Beadchip, the study revealed that long-term exposure to wildfire-related PM2.5 (>3 years) was associated with differences in blood DNA methylation of 26 CpGs and 33 differentially methylated regions (DMRs). These genomic regions were mapped to 47 genes enriched for pathways related to inflammatory regulation and platelet activation, and were linked to various human diseases and phenotypes, including cancer, mental disorders, diabetes, obesity, asthma, and blood pressure. This study underscores the potential for wildfire smoke exposure to induce epigenetic changes, which may contribute to the development of mental health problems. Understanding these mechanisms is crucial for developing targeted interventions to mitigate the adverse mental health effects of wildfire exposure.

### Hurricane

Kello et al. (2023) investigated hurricane Maria, which was one of the largest natural disasters to affect Puerto Rico. The authors reported that 47 significant differentially methylated probes (DMPs) were associated with various hurricane-related variables, with 30 significant DMPs specifically linked to the gestational stage at the time of the hurricane. Most of the DMPs associated with the timing of exposure were hypermethylated, and the most significant clusters were located on chromosomes 1–4. Additionally, maternal mental status and property damage following the hurricane were associated with notable variations in DNA methylation patterns. These findings suggest that both the timing of prenatal exposure and post-hurricane stressors may influence DNA methylation, potentially affecting long-term health outcomes.

### Ice storm

A study investigating genome-wide DNA methylation levels in adolescents found that maternal objective hardship led to methylation changes in genes involved in immune function, whereas maternal subjective distress did not impact genome-wide methylation profiles (Cao-Lei et al., 2014). Using blood samples from 13-year-olds, T cell DNA was analyzed with the Illumina HumanMethylation450K Beadchip, revealing comparability of methylation profiles across T cells, saliva, and peripheral blood mononuclear cells (PBMCs). Additionally, maternal cognitive appraisal was linked to methylation changes in immune-related genes (Cao-Lei et al., 2015). The overlap in genes and biological pathways associated with cognitive appraisal and objective hardship suggests distinct yet interconnected pathways through which different aspects of prenatal stress affect methylation.

### Earthquake

Wang et al. (2022) investigated the association between prenatal exposure to stress caused by the 1976 Tangshan earthquake in China and the methylation of CpG sites within the NR3C1 exon 1F promoter. The researchers also explored how these epigenetic modifications may impact working memory in adulthood. The findings indicated that individuals prenatally exposed to the earthquake exhibited significantly higher NR3C1 methylation levels compared to those who were not exposed to such stress. Notably, among those exposed, individuals in the second trimester showed significantly higher methylation levels than those in the third trimester. Additionally, impaired working memory was observed in subjects exposed to prenatal earthquakes during the second trimester. The study found a moderate negative correlation between methylated CpG1 in the NR3C1 exon 1F promoter and working memory specifically in the second trimester group, suggesting that CpG1 methylation may play a role in the relationship between earthquake-related prenatal stress and long-term effects on working memory. Moreover, a study from the OBESO perinatal cohort in Mexico City investigated the impact of acute gestational stress from the 2017 earthquake on mitochondrial DNA copy number (mtDNAcn) (Mendoza-Ortega et al., 2021). The researchers compared mtDNAcn in umbilical cord blood from infants born before the earthquake, those whose mothers were pregnant during the earthquake, and those conceived after the earthquake. Using quantitative real-time PCR, they found that newborns exposed to the earthquake *in utero* or conceived afterward had significantly higher mtDNAcn than those not exposed, suggesting mtDNAcn as a potential biomarker for acute stress. The study emphasizes the importance of long-term monitoring of children born to mothers who experienced prenatal stress, especially from natural disasters.

## The potential role of epigenetics in prevention and intervention

Integrating epigenetic research into therapeutic and preventive strategies for mental health offers promising opportunities to develop personalized interventions aimed at mitigating the adverse effects of climate change on mental wellbeing. Epigenetic markers act as accessible and dynamic biosensors, capturing both biological and biographical risks for mental disorders, making them valuable not only as indicators but also as targets for preventive measures (Domschke, 2021). Musci and Schlomer (2018) emphasize the importance of including genetic and epigenetic research in preventive medicine, arguing that understanding these factors allows for the creation of individualized interventions that can prevent the onset of mental disorders.

Domschke (Domschke, 2021) further suggests that targeting epigenetic markers in prevention could foster resilience against mental disorders, potentially halting their transmission to future generations. This concept of “epigenetic memory” for environmental adaptations is particularly relevant given evidence that epigenetically imprinted trauma can be passed across generations through the germline (Bohacek and Mansuy, 2015). Such findings raise the possibility of “transgenerational prevention” where successful interventions embodied in epigenetic signatures could equip future generations with an enhanced ability to adapt to environmental stressors, including those linked to climate change.

Moreover, research by Hong and Efferth (2016) demonstrated a link between epigenetic modifications and PTSD risk and memory function in survivors of the Wenchuan earthquake, highlighting the need for further studies on patient-specific criteria, social support roles, and alternative treatments such as Chinese medicine. Olson et al. (2019) also stress the need to explore the epigenetic changes triggered by natural disasters, particularly in terms of adverse effects on pregnancy and fetal development, which could have lasting, transgenerational impacts.

By deepening our understanding of the genetic and epigenetic mechanisms underlying mental health disorders and their environmental triggers, more effective strategies can be developed to build resilience and prevent the transmission of these disorders across generations. Future research should prioritize early interventions, policy integration, and public health strategies that address the large-scale risk factors posed by climate change-related disasters, paving the way for more robust mental health prevention and intervention efforts.

## Perspectives

Despite significant strides in understanding the impacts of climate change-related disasters on mental health and epigenetic modifications, several critical gaps persist in the current literature.

### Sex effect

The influence of climate change on natural disasters cannot be overlooked, as it directly and indirectly impacts the occurrence of such events. Above-mentioned studies have revealed that various disasters have an impact on women more than men, and women are more susceptible to experiencing poor sleep quality, depression disorders, mood disorders, and PTSD in comparison to men (Alfuqaha et al., 2023; Ataya et al., 2024; Chen et al., 2023b; Han, 2023; Hikichi et al., 2023a; Lu et al., 2023). Research indicates that biological sex can influence susceptibility to stress and subsequent epigenetic alterations, yet studies often do not disaggregate findings by sex. Future research should systematically examine how sex differences influence the manifestation of epigenetic changes and mental health responses.

### Vulnerable populations

Climate shifts have increased the range and seasonality of vector-borne diseases, creating global public health challenges and affecting vulnerable populations (Romanello et al., 2021). Children are more likely to be susceptible to the health impacts of climate change-related disasters, such as respiratory issues, heat stress, and infectious diseases (Romanello et al., 2021). With their developing bodies, children breathe more air, drink more water, and spend more time outdoors relative to their body weight, making them more susceptible to environmental hazards like air and water contamination (Makharia et al., 2023). Older adults face greater risks from reduced physiological resilience and often pre-existing health conditions. There is increased sensitivity to environmental changes and exposures as a by-product of lowered physiological reserve capacity, slower metabolism, and a slower immune system (Carnes et al., 2014). Moreover, low-income communities are disproportionately affected by the health and environmental consequences of climate change (Levy and Patz, 2015). They are more likely to be exposed to environmental hazards without the proper resources to reduce the impact. People with lower socioeconomic status are more likely to live in regions highly exposed to the risk of climate change (e.g., flood-prone regions), areas with poor air quality, or near industrial sites, which negatively impact health (Makharia et al., 2023). Furthermore, Indigenous communities around the world continue to face political, economic, and racial marginalization, further burdened by the effects of climate change (Redvers et al., 2023). Environmental changes from climate change, such as the loss of traditional lands and ecosystems, were found to significantly impact the mental health of Indigenous peoples living in their traditional territories. It included acute psychological stress from environmental changes, climate-related disasters, and cultural and spiritual dislocation due to the loss of traditional lands and practices (Grande et al., 2023). It heightened social and economic pressures that exacerbate existing health inequalities. Despite these challenges, Indigenous communities show resilience, using their knowledge and social networks to adapt to the adverse effects of climate change. Climate policies and interventions should understand the Indigenous perspectives and needs, implementing culturally tailored mental health services and support systems. Therefore, more attention should be given to the unique experiences and coping mechanisms of vulnerable populations in the face of climate change in order to develop effective strategies that prioritize their wellbeing and sustainability.

### Combined effects of disasters

The difficulty of studying the combined effects of multiple disasters, such as extreme temperatures and pandemics, presents significant challenges in understanding and addressing their mental health and epigenetic impacts. The occurrence of disasters, like those observed during the COVID-19 pandemic, exacerbates the complexity of these effects. In addition, according to the 2021 IPCC report, it is anticipated that as global warming progresses, extreme temperatures will become more frequent. All data models indicate a 1.5-degree Celsius rise in global temperatures by 2050, which will likely increase the frequency and intensity of heatwaves and other extreme weather events (2021). Recent studies underscore this trend, revealing a significant increase in the frequency of heatwaves since the mid-twentieth century, particularly in regions already vulnerable to climate change (Perkins-Kirkpatrick and Lewis, 2020). Additionally, research by Sanches et al. observed a rise in the intensity and frequency of surface air temperature extremes along the western South Atlantic coast over the past 40 years, further highlighting the escalating threat posed by extreme temperatures (Sanches et al., 2023). Addressing the mental health and epigenetic impacts of these climate change-induced disasters, especially in the context of concurrent events, requires a multifaceted approach. Research must consider the interplay between various stressors and their cumulative effects on mental health and epigenetic modifications. This holistic understanding is crucial for developing effective intervention strategies to mitigate the adverse health impacts of climate change-related disasters.

### Potential mediating role of epigenetics

As previously discussed, we have provided evidence demonstrating how these climate change-related disasters influence both mental health outcomes and epigenetic changes. Epigenetic mechanisms, particularly DNA methylation, offer a dynamic system by which environmental factors, such as stress induced by natural disasters, can regulate gene expression and contribute to long-term psychological impacts.

Recent studies have highlighted the potential of DNA methylation as a significant contributor to various mental disorders, including depressive disorder, panic disorder, autism spectrum disorder (ASD), attention deficit hyperactivity disorder (ADHD), and borderline personality disorder. For instance, Barbu et al. (2021) established a Methylation Risk Score (MRS) for Major Depressive Disorder (MDD), which was correlated with both current and future depression. This score demonstrated associations with lifestyle factors, such as smoking and alcohol use, both of which are known to influence DNA methylation and contribute to depression (Barbu et al., 2021). In line with these findings, Czamara et al. (2022) investigated the relationship between DNA methylation, panic disorder (PD), and MDD in response to stressful life events. Their results showed that gene expression alterations in PYROXD1 and GFOD2 were significantly associated with PD and MDD (Czamara et al., 2022). Furthermore, the InterGEN study involving Black women provided evidence that psychosocial stressors could induce alterations in DNA methylation, which were linked to depressive symptoms. The study identified several genes, including GLRX5, CLEC1B, and NBPF8, among others, that were associated with depressive symptoms and neurological diseases (Taylor et al., 2024). Additional research has also supported the role of epigenetics in mediating mental health outcomes related to other environmental stressors. Chrétienneau et al. (2024) found distinct DNA methylation patterns in the OXTR, CRH, and NTF3 genes in individuals with substance use disorders (SUD) who had experienced paternal abuse, linking these patterns to an elevated risk of suicidal behavior (Chrétienneau et al., 2024). Similarly, Zhu et al. (2022) demonstrated that placental DNA methylation of the NHIP gene is associated with increased ASD risk in high-risk cohorts (Zhu et al., 2022). Feil et al. (2023) also explored the relationship between air pollution and neurodevelopmental delays, showing that DNA methylation in genes such as GOPC and DYRK1A mediated the effects of air pollution on cognitive development (Feil et al., 2023). Another noteworthy example is the study by Fotopoulos et al. (2024), which combined neuroimaging and epigenetics to investigate the impact of prenatal smoking on children with ADHD. The findings revealed significant reductions in cortical surface area in brain regions linked to attention regulation and impulsivity, suggesting that epigenetic markers can be used to assess prenatal smoking exposure and its subsequent effects on brain development and ADHD (Fotopoulos et al., 2024).

These findings provide evidence that epigenetic modifications, particularly DNA methylation, could mediate the impact of various environmental stressors on mental health. As research in this field continues to evolve, the role of epigenetics in linking environmental factors, such as climate change-related disasters, to mental health outcomes becomes increasingly clear. Future research should focus on expanding our understanding of these epigenetic pathways and identifying potential therapeutic targets for mitigating the mental health consequences of such environmental stressors.

### Other mechanisms of epigenetics and the need for longitudinal studies

While much of the current research has focused on DNA methylation, it is essential to recognize that other epigenetic mechanisms, such as histone modifications and non-coding RNAs, are also crucial regulators of gene expression. These mechanisms may play significant roles in mediating responses to environmental stressors, including those related to climate change. To gain a more comprehensive understanding of the long-term mental health impacts of such stressors, future research should expand beyond DNA methylation and explore the broader epigenetic landscape. This includes investigating histone modifications, chromatin remodeling, and non-coding RNA interactions, all of which may contribute to the complex regulatory networks that influence mental health.

Moreover, there is a pressing need for longitudinal studies that track individuals over extended periods to capture the dynamic nature of epigenetic changes and their interaction with genetic and environmental factors. Longitudinal designs can reveal whether initial epigenetic modifications persist or evolve in response to ongoing stressors, potentially serving as biomarkers for long-term mental health risks exacerbated by climate-related disasters. These studies should also consider developmental windows of vulnerability, examining how disaster exposure shapes epigenetic profiles and mental health trajectories across the lifespan. By monitoring these changes over time, researchers can identify critical periods of susceptibility and resilience, informing the development of more targeted and personalized interventions to mitigate the adverse mental health effects of climate-related stress.

Integrating multi-omics approaches into longitudinal studies will further enhance our understanding of how various epigenetic mechanisms contribute to mental health outcomes, ultimately paving the way for more effective prevention and intervention strategies in the context of environmental stress.

## Conclusion

The review underscores the complex interplay between climate change-related disasters, mental health outcomes, and epigenetic modifications. While much of the current research has focused on DNA methylation, it is crucial to explore other epigenetic mechanisms, such as histone modifications and non-coding RNAs, to gain a more comprehensive understanding of how these mechanisms mediate the long-term mental health effects of environmental stressors. The evidence suggests that epigenetic markers serve not only as indicators of mental health risks but also as potential targets for personalized preventive interventions, which may help build resilience against mental disorders and mitigate their transmission to future generations. However, significant gaps remain in the literature, particularly regarding the combined effects of multiple disasters and the long-term impact of these events on epigenetic changes. Future research should focus on integrating multi-omics approaches, conducting longitudinal studies, and developing tailored interventions that address the unique needs of vulnerable populations. By advancing our understanding of these epigenetic pathways, we can create more effective strategies for preventing and mitigating the mental health consequences of climate change-related disasters.
